# Understanding Impact of Anti‐Obesity Medications on Skeletal Muscle Mass Change Is Confounded by Measurement Methods

**DOI:** 10.1111/obr.70041

**Published:** 2025-11-24

**Authors:** Arden McMath, Dympna Gallagher

**Affiliations:** ^1^ New York Nutrition Obesity Research Center Columbia University New York City USA; ^2^ Division of Endocrinology, Department of Medicine Columbia University Irving Medical Center New York City USA; ^3^ Institute of Human Nutrition Columbia University New York City USA

**Keywords:** body composition, glucagon‐like peptide‐1 receptor agonists, obesityoverweightanti‐obesity medicationsskeletal muscle mass

## Abstract

Anti‐obesity medications promote greater degrees of weight loss than lifestyle interventions alone. There is an important need to understand whether loss of skeletal muscle during pharmacologically induced weight loss is clinically significant due to its essential role in health and disease. Most randomized, placebo‐controlled studies addressing this question report on fat‐free or lean mass estimated from dual‐energy x‐ray absorptiometry or bioelectrical impedance without defining the composition of these components. Fat‐free, lean, and skeletal muscle mass are not synonymous terms, and studies frequently fail to define lean mass, which may or may not include bone. Lack of standard preparatory procedures prior to measurement, differences in medications, doses, or intervention lengths, and inclusion of varied lifestyle modifications prevent reaching a consensus regarding the impact on skeletal muscle of pharmacologically induced weight loss. There is a critical need for greater precision and depth of understanding when selecting a measurement method and describing body compartments.

**Abbreviations**
AOM: anti‐obesity medication
BIA: bioelectrical impedance
BMD: bone mineral density
CT: computed axial tomography
DXA: dual‐energy x‐ray absorptiometry
FFM: fat‐free mass
GLP‐1 RAs: glucagon‐like peptide‐1 receptor agonist
LM: lean mass
LST: lean soft tissue
MRI: magnetic resonance imaging
REE: resting energy expenditure
SMM: skeletal muscle mass
TBW: total body water
ULM: undefined lean mass

## 1 Introduction

Pharmacological approaches to weight loss frequently promote greater weight loss compared to lifestyle interventions involving diet and physical activity alone [1, 2]. With the rapid rise in the use of currently approved anti‐obesity medications (AOMs) and the development of many new AOMs on the horizon, it is important to consider potential unintended clinical consequences of their use. Understanding the composition of body mass lost is crucial to fully elucidate AOMs' role in the treatment of overweight and obesity. Whether AOMs cause disproportionate losses in the skeletal muscle mass (SMM) component of the body's fat‐free mass (FFM) is unclear [3, 4, 5, 6]. This review critically examines existing evidence regarding changes in SMM during pharmacologically induced weight loss.

## 2 SMM Function and Quantification

SMM is vital to movement, posture, and activities of daily living. SMM aids in maintaining body temperature and nutrient storage. SMM is composed primarily of amino acids, which can be repurposed for the synthesis of new proteins during recovery from acute injury or illness. The clinical significance of adequate amounts of SMM is evident in individuals with obesity, as those with adequate SMM have a lesser risk of death, comorbidities, metabolic disorders, and geriatric syndromes compared to those with obesity plus sarcopenia (e.g., age‐related low SMM) [7].

SMM quantity and proportion relative to body weight depend on factors such as age, sex, body mass, height, race, physical activity, and diet [8]. SMM quantified via whole‐body magnetic resonance imaging (MRI) in a large, heterogeneous, cross‐sectional sample of 468 adults with normal or overweight demonstrated that adult females and males had an average of 31% and 38% SMM relative to their total body weight. At ages below 40 years, lower relative SMM is attributed to greater body fat, rather than low SMM [9]. Evidence suggests that absolute losses in SMM begin after approximately age 45 years, after which 1.9 and 1.1 kg of SMM is lost per decade in males and females, respectively [9]. Individuals who are taller and with greater adiposity require greater SMM (especially lower body SMM) to move their larger mass. With excess weight gain, the proportional increase in SMM is smaller, so those with overweight and obesity have lower SMM relative to body weight [9].

Notably, in vivo body composition measurement methods all rely on estimation since body tissues cannot be removed for weighing. At the tissue‐organ‐systems level of body composition, the body can be partitioned into bone and soft tissues, where soft tissues primarily include adipose tissue and skeletal muscle [10]. Volume measurements can be converted to mass using conversion factors based on assumed densities (e.g., 1.04 for skeletal muscle and 0.92 for adipose tissue) [11, 12]. MRI and CT are considered the more accurate methods [13], but their use is limited due to high expense, accessibility, and, for CT, radiation exposure. Other available methods of estimating SMM include D3 creatine dilution or urinary creatine, which are limited due to expense, logistical burden, or inaccessibility in clinical or research settings [14, 15].

**FIGURE 1:**
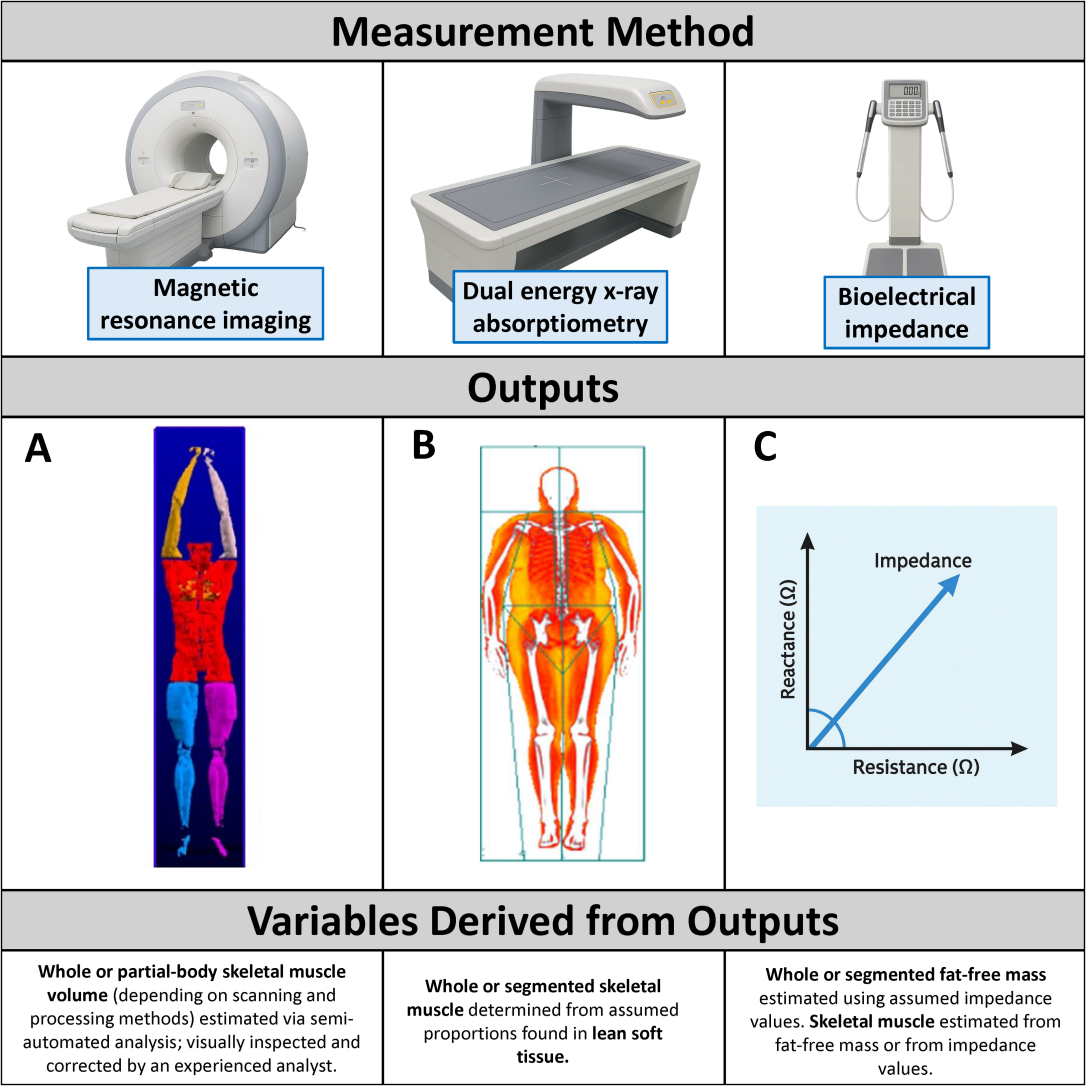
Outputs and variables derived from outputs from methods that have been used for estimating fat‐free mass and skeletal muscle mass in studies involving AOM. (A) Three‐dimensional reconstruction of whole‐body skeletal muscle from a contiguous magnetic resonance imaging scan, from Shen et al. with permission [16] (note, this imaging approach includes neck and below the knee, whereas Neeland et al. [17] only included neck‐to‐knee skeletal muscle quantification). Image is color‐coded by body compartments: left arm (tan), right arm (yellow), trunk and neck (red), left leg (purple), and right leg (blue). (B) Image is from GE iDXA total body scan in the Body Composition Unit of the New York Nutrition Obesity Research Center. Image is color‐coded by tissue type: bone (white), fat tissue (yellow), and lean soft tissue (red). (C) Image created to show result of impedance comes from reactance and resistance.

Most studies reporting on body composition changes using AOMs have used methods that target molecular levels of body composition assessment, such as bioelectrical impedance (BIA) or dual‐energy x‐ray absorptiometry (DXA). BIA and DXA provide estimates of FFM (Figure 1), where FFM is the difference between body weight and fat mass. Fat mass in this context is made up of essential and nonessential lipids, regardless of where they reside in the body. FFM therefore consists of proteins, minerals (including bone), total body water (TBW), and small amounts of essential lipids (e.g., cell membranes or organelles) [10, 18]. DXA cannot resolve soft‐tissue composition within bone pixels, causing lipid within the marrow of bones to modestly influence bone mineral density (BMD) and, by extension, the FFM component since it includes bone [19, 20].

Compared to FFM, lean soft tissue (LST) is closer to but not synonymous with SMM and can be derived from these methods through subtraction of estimated bone mass from the FFM component [14, 21]. DXA‐derived LST and LST outputs trained against DXA standards for bone masses could also theoretically be influenced by lipids in bone marrow, although the extent to which this occurs is unknown. BIA itself does not estimate bone mass. Regardless, LST measured via DXA or BIA contains protein, minerals estimated to reside outside bone, TBW, and some essential lipids. Relevant to the interpretation of LST and FFM changes during weight loss, proteins estimated via these methods are derived from all muscle tissues, namely, skeletal, smooth (e.g., digestive tract), and cardiac muscle, as well as other tissues containing protein, such as tendons, ligaments, and organs [10, 22]. The term “muscle mass” should not be confused with SMM, as the former contains smooth and cardiac muscles [10]. Relevant to the interpretation of fat mass changes during weight loss, lipids estimated via these methods reside both inside and outside adipose tissue. Adipose tissue includes lipids, connective tissue, and small amounts of water [10, 23].

**FIGURE 2:**
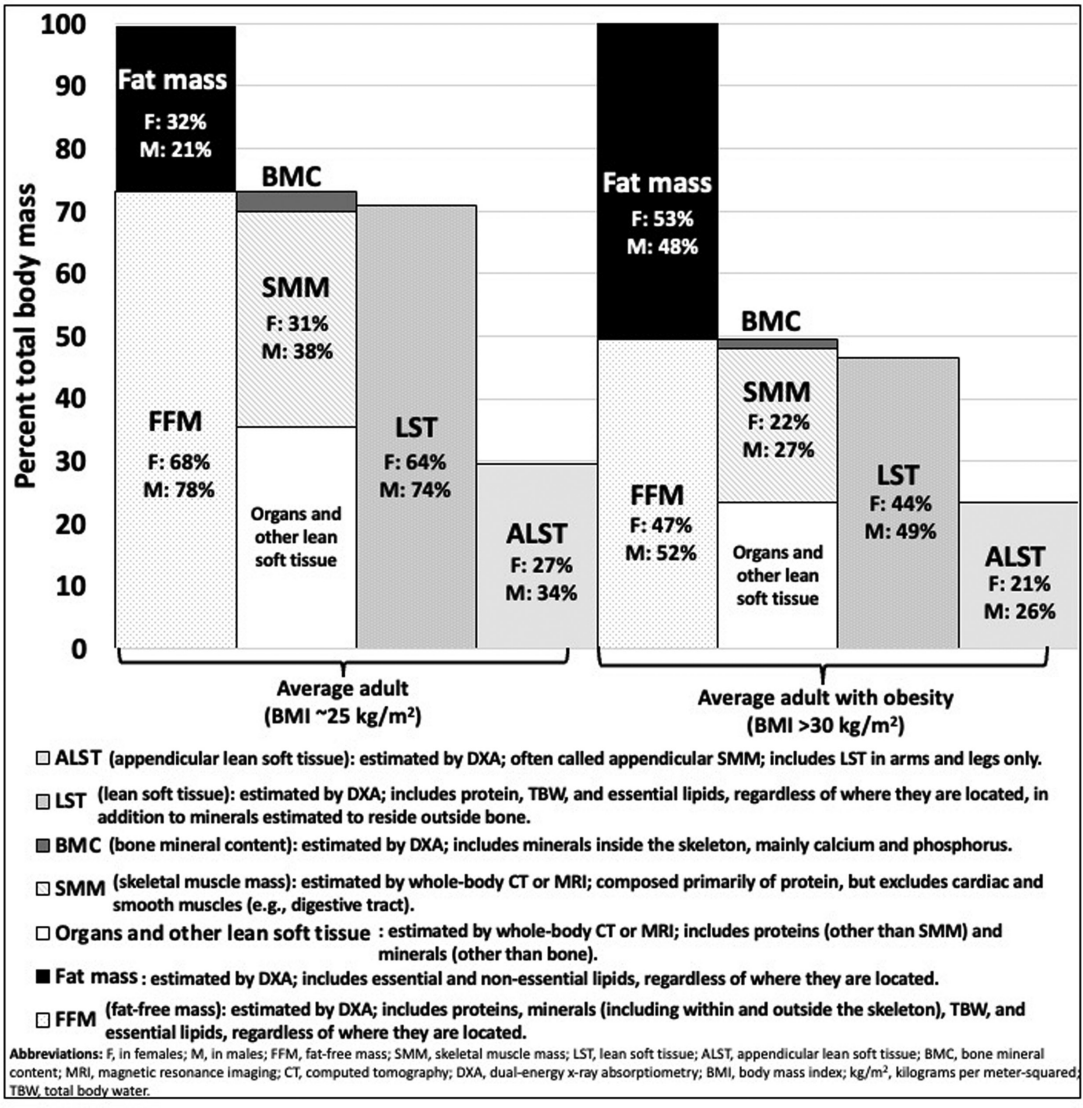
Proportion of body weight represented by fat‐free mass, skeletal muscle mass, lean soft tissue, and appendicular skeletal muscle mass in adults with average BMI (24–25 kg/m^2^) [9, 24, 25, 26] and obese BMI [27, 28]. Proportions represent average of values between males and females.

Estimated proportions of these components for adults with average BMI (25 kg/m^2^) [9, 24, 25, 26] and obese BMI (> 30 kg/m^2^) [27, 28] are shown in Figure 2. SMM is the largest component of FFM (Figure 2), accounting for 45%–50% of total FFM in various populations with healthy and overweight [29, 30, 31, 32], and 47% and 52% of FFM was SMM in females and males with obesity, respectively [27]. Appendicular LST (ALST) derived from DXA, which represents the nonfat and nonbone mass in arms and legs, makes up ~42% of FFM and more closely represents SMM compared to whole‐body FFM [26]. ALST derived from DXA and BIA is sometimes referred to as appendicular SMM, which is misleading since this component represents the quantity of all lean tissues within the appendages (e.g., protein‐containing connective tissues) and not just skeletal muscle. SMM increases as body mass increases in a curvilinear manner [31]. These extrapolations of FFM or LST to SMM introduce variability in accuracy and precision (Table 1) [14, 15, 42].

**TABLE 1:** Methods and sources of potential bias for estimation of changes in skeletal muscle mass with use of anti‐obesity medications.

Method	If applicable, proxy used for skeletal muscle mass	Mechanism	Precision error^a^	Potential sources of error
Bioelectrical impedance	Fat‐free mass	Resistance and reactance are measured from a single (single frequency bioelectrical impedance analysis), multiple (multifrequency bioelectrical impedance analysis), or range of frequencies (bioelectrical impedance spectroscopy) of electrical currents as they pass through the body via body water; with height and weight of the person, body composition is estimated using measured resistivity [33].	Single frequency bioelectrical impedance: Prediction of total body water is within 3.6 and 2.6 L for males and females, respectively. Prediction of fat‐free mass is within 3.7 and 2.8 kg for males and females, respectively Multiple frequency bioelectrical impedance: Total body water is within 1.3 kg. Fat‐free mass is within 1.9 kg [34].	Sources of error include the following: (1) All body tissues and fluid pools are assumed to have the same resistivity, (2) the hydration fraction of the fat‐free mass is assumed most often to be 0.73, and (3) electrolyte status is assumed normal. (4) Validated prediction equations using resistance and reactance are used to estimate fat‐free mass and total body water, (5) single‐frequency BIA is limited because (a) it cannot pass through cell membranes and thus assess intracellular water (included in skeletal muscle cells), and (b) many models measure from one point to another (usually wrist to ankle) rather than separating the body into parts (i.e., arms, legs, and trunk), (6) assumes that adipose tissue is relatively anhydrous; when amount of adipose tissue is small, this may apply. In persons with obesity and following significant weight loss involving large amounts of adipose tissue, the water contribution may not be negligible [33, 34, 35].
Dual‐energy X‐ray absorptiometry	Fat‐free mass	Difference in absorption of two different energy X‐rays is used to measure soft (nonbone fat and lean) versus hard (bone mineral) content, due to the distinct attenuation properties of these tissues. A proprietary algorithm estimates fat and fat‐free mass within the measured soft tissue.	Fat‐free mass is within 1.6 kg [36]	Sources of potential bias include the following: (1) A hydration fraction of 0.73 for fat‐free mass is assumed, (2) tissue thickness impacts accuracy of fat and fat‐free mass measurement due to beam‐hardening errors, and (3) DXA cannot measure soft tissue within bone pixels, which impairs ability to determine fat and nonbone lean tissue in regions that contain bone (i.e., pelvis, abdomen, and trunk), (4) assumes that adipose tissue is relatively anhydrous; when amount of adipose tissue is small, this may apply. In persons with obesity and following significant weight loss involving large amounts of adipose tissue, the water contribution from it may not be negligible [37, 38]
Neck‐to‐knee magnetic resonance imaging using AMRA approach	Neck‐to‐knee skeletal muscle mass	A magnetic field is used to align all hydrogen proton axes in the body and oriented along the axis of the MRI scanner. This is called a magnetic vector. This vector is pulsed with radio frequency, causing the vector to deflect. When radio frequency pulses are discontinued, the protons return to their resting state, releasing additional radio waves that are used in the creation of hydrogen proton magnetic resonance images. These images are separated by water and fat [39]. This describes the image acquisition and reconstruction steps, where other steps include segmentation and tissue quantification. AMRA differs from other MRI processing approaches in that it automates segmentation and quantification using the signal generated from fat and the comparison of images to a library of manually segmented images [40].	Neck‐to‐knee skeletal muscle mass precision data for AMDR approach is not available; only regional precision data available (see below).	Due to variation in radio frequency emissions from protons that exist between individuals and slices, pixels cannot be assigned to a given tissue using a single threshold. Manual verification/correction is necessary and introduces a degree of subjectivity [41].
Magnetic resonance imaging of thigh using AMRA approach	Skeletal muscle volume of thigh		Skeletal muscle volume of the thigh was within 0.06 L or 0.06 kg^104^ ^b^	All of the above sources of error apply, in addition to error from the prediction of whole‐body skeletal muscle from single or multiple slices rather than whole body.

^a^ Precision errors are device‐specific and serve to provide an approximate example of the precision error of the measurement method.

^b^ Factor of 1.04 used to convert volume (liters) to mass (kilograms).

The term “lean mass” (LM) is frequently reported from studies using DXA and BIA and could refer to the LST plus bone mass, which is the equivalent of FFM. LM can also refer to the LST, which does not include bone. Some studies state that bone mass is included, while others do not when using the term “LM” [43]. For brevity, the term LST will be utilized herein where it can be differentiated from FFM (does not include bone). In cases where it cannot be determined whether it is FFM or LST, the term “undefined lean mass” (ULM) will be used.

## 3 Weight Loss Effects on SMM

Weight loss reflects reductions of both fat mass *and* FFM. An important question is whether the proportion of body mass, that is, SMM, changes from before to after weight loss. The amount of FFM and the SMM component of FFM lost during weight loss is influenced by participant‐specific and weight loss intervention characteristics. Postmenopausal women, older adults, and those with chronic diseases are especially susceptible to a greater loss of SMM [44].

While most weight loss interventions adequate in protein yield a greater percent fat than FFM loss, it is clinically important to understand the effects of SMM loss for optimal physical functioning, maintenance of reduced weight, and metabolic health. In a meta‐analysis including 1063 individuals with overweight or obesity and related comorbidities, those who achieved greater FFM retention during weight loss exhibited a greater fat mass loss, greater reductions in blood triglycerides, and less reduction in resting energy expenditure (REE) [45]. Excessive losses in SMM may also contribute to the inability to maintain the weight‐reduced state, as SMM is a large contributor to REE. Those with higher SMM loss are likely to have greater reductions in the already established lower REE post‐weight loss [46], making it difficult to remain in energy and thus weight balance.

The quantity and quality of SMM and metabolic health of individuals prior to weight loss may serve to guide obesity treatment decisions. A recent study found that individuals with overweight or obesity, and without diabetes but with lower insulin sensitivity at baseline, lost more LST [47]. Selecting a pharmacological intervention that minimizes losses of SMM or provision of recommendations to increase SMM before attempting weight reduction in individuals with sarcopenic obesity or metabolic derangements is likely to minimize risks and maximize benefits associated with weight loss. Small losses of SMM during weight loss could be detrimental to individuals if their amino acid pool is already sparse (i.e., sarcopenic obesity) [48].

Loss of SMM during diet‐induced weight loss is highly dependent on intervention length, degree of caloric restriction, and protein intake. In healthy young‐to‐middle‐aged persons without obesity (21–50‐years old; BMI of 22–28 kg/m^2^), ~13.7% calorie restriction over 2 years resulted in 11.6% weight loss, of which 17.2% was SMM and 3.5% was residual lean tissue (including bones, tendons, connective tissue, heart, and digestive tract) assessed by whole‐body MRI. By contrast, controls consuming an ad libitum diet over the 2 years gained 2.0 kg and did not lose a significant amount of SMM. The loss of SMM increased with greater degrees of calorie restriction [25]. Dietary interventions employed in persons with overweight or obesity reported 16%–33% of total weight loss from FFM (1–3.2 kg) [49, 50, 51, 52] and 12%–23% from SMM (0.9–1.7 kg) [53, 54]. Dose‐dependent FFM retention can be achieved through increases in dietary protein intake during weight loss interventions. However, FFM losses are not completely abrogated [55], and protein intakes as high as threefold the recommended daily allowance may be required to attenuate endogenous protein breakdown that occurs during clinical weight loss trials [56]. Addition of aerobic and resistance exercise attenuates losses of FFM [44] and may even preserve age‐related SMM loss from caloric restriction [57]. Studies that incorporated exercise with caloric restriction reported FFM losses between 16% and 21% of total weight lost [51, 53, 58].

## 4 Studies Assessing Change in Measures of SMM During AOM Use

This review is narrative in nature and does not follow the procedures of a formal systematic review. To enhance applicability, transparency, and reproducibility, a semi‐structured search and selection process was employed to identify literature for critical review of methods. Using a predetermined search strategy (Table S1), PubMed was searched for studies most relevant to changes in SMM during AOM use. Eligible studies included randomized placebo‐controlled trials (RCTs) of adults with overweight or obesity that reported the change in a variable called “LM” (Table 2), FFM (Table 3), and SMM or muscle mass (Table 4) during weight loss involving AOMs. Studies included those with and without diabetes or other chronic diseases. Studies involving children, animals, in vitro, or phase I trials were excluded. One author (AM) searched PubMed using the search strategy designed a priori for this review. AM selected articles for inclusion/exclusion based on predetermined criteria outlined above. The corresponding author (DG) confirmed the relevance of studies included by AM before literature synthesis. No formal quality assessments were employed. Instead, strengths and limitations relevant to SMM estimation and determining the clinical significance of SMM changes are discussed.

**TABLE 2:** Studies reporting impact of FDA‐approved anti‐obesity medications on loss of compartment termed “lean mass,” which was not described and may (same as fat‐free mass) or may not (same as lean soft tissue) include bone.

Body composition measurement	Study	Total *N* (*N* intervention group(s))	Anti‐obesity medication, dosage(s), and frequency	Exercise (E) or diet (D) intervention^a^	Intervention length (weeks)	Weight change (kg)	kg Change in lean mass (% total weight lost) unless noted	Study design and comments
Neck‐to‐knee magnetic resonance imaging	Neeland et al., 2021 [59]	185 (92)	3.0‐mg/d liraglutide	E + D	40	−6.8	−0.54 L or −0.56 kg^b^ (8.2)	Randomized, double‐blind, placebo‐controlled study design. Reported lean tissue volume only, despite mentioning “muscle” in methods.
BIA model InBody 230 multifrequency analyzer (Biospace Co. Ltd.)	Moldovan et al., 2016 [60]	87 (44)	37.5‐mg/d phentermine HCl	E + D	12	−13.4	−2.1 (15.7)	Randomized, double‐blind, placebo‐controlled study design. Term used, lean body mass, does not match output variables (FFM or SMM) in InBody 230 manual. Two frequencies are used, 20 and 100 kHz. Estimates TBW only [61].
DXA make Hologic, model not specified	Tardio et al., 2024 [62]	82 (56)	Extended release 16‐ and 32‐mg naltrexone plus 360‐mg bupropion (two groups, results pooled)	—	52	−7.2^c^	−2.3 (4.1)^c^	Multicenter, randomized, double‐blind, placebo‐controlled study design. Term used was lean mass. Compartment not directly defined but is likely FFM, since total body mass was described as lean mass plus fat mass in the text.
DXA model Hologic 4500 (Waltham, MA)	Tuccinardi et al., 2019 [63]	48 (24)	10‐mg 2×/d lorcaserin	—	24	−8.2	−4.1 (50.0)	Randomized, double‐blind, placebo‐controlled study design.
DXA model not specified	Fidler et al., 2011 [64]	2224 (1390)	10‐mg 2×/d lorcaserin	E + D	52	−5.8	−0.91 (15.7)^d^	Multi‐site, randomized, double‐blind, placebo‐controlled, parallel group study design.
			10‐mg 1×/d lorcaserin			−4.7	−0.94 (20.0)^d^	
DXA model Hologic Discovery A version 13.4.2 (Hologic Inc., Waltham, MA)	Mok et al., 2023 [65]	70 (35)	3.0‐mg/d liraglutide	E + D	24	−9.5	−3.2 (33.7)	Randomized, double‐blind, placebo‐controlled, parallel group study design.
DXA model Hologic Discovery A model (Hologic Inc., Waltham, MA)	Elkind‐Hirsch et al., 2022 [66]	82 (55)	3.0 mg/d liraglutide	E + D	32	−6.3	0.0 (0.0)	Randomized, double‐blind, placebo‐controlled study design.
DXA model DPX–IQ Lunar (General Electric Lunar Corporation, Madison, WI)	Mensberg et al., 2016 [67]	34 (18)	1.8‐mg/d liraglutide	E	16	−3.4	−0.1 (2.9)	Randomized, double‐blind, placebo‐controlled study design.
DXA model Hologic Discovery A (Hologic Inc., Marlborough, MA)	Schmidt et al., 2021 [68]	44 (22)	1.8‐mg/d liraglutide	—	26	−7	−2.5 (35.7)	Dual‐site, randomized, double‐blind, placebo‐controlled study design.
DXA model not specified	Ghanim et al., 2020 [69]	84 (42)	1.8‐mg/d liraglutide	—	26	−4.2	0.0 (0.0)	Randomized, double‐blind, placebo‐controlled study design.
DXA model Hologic Discovery (Bedford, MA)	Frossing et al., 2017 [70]	72 (48)	1.8‐mg/d liraglutide	—	26	−5.2	−2.4 (46.2)	Randomized, double‐blind, placebo‐controlled study design.
DXA model Lunar Radiation (Madison, WI)	Harder et al., 2004 [71]	33 (21)	0.6‐mg/d liraglutide	—	8	−0.7	+0.7 (0.0)	Randomized, double‐blind, placebo‐controlled, parallel group study design.
DXA model not specified	Jendle et al., 2009 [72]	123 (103)	0.6‐mg/d liraglutide + metformin	—	26	−0.9	−0.3 (33.3)	Randomized‐controlled trial, double‐blind, double‐dummy, placebo‐controlled study design.
			1.2‐mg/d liraglutide + metformin			−2.0	−0.8 (40.0)	
			1.8‐mg/d liraglutide + metformin			−3.2	−1.5 (46.9)	
DXA model not specified	Jendle et al., 2009 [72]	61 (43)	1.2‐mg/d liraglutide	E + D	52	−2.4	−1.1 (45.8)	Randomized‐controlled trial, double‐blind, double‐dummy, placebo‐controlled study design.
			1.8‐mg/d liraglutide			−2.3	−1.5 (65.2)	
DXA models Hologic or Lunar, depending on site	Jastreboff et al., 2022 [17]	2539 (1896)	5‐mg/wk tirzepatide	E + D	72	−16.1	~−4.8 (23.2)^e^	Randomized, double‐blind, placebo‐controlled study design. Only a subset of participants (*n* = 160) had body composition assessed.
			10 mg/wk tirzepatide			−22.4		
			15‐mg/wk tirzepatide			−23.6		
DXA model not specified	Wilding et al., 2021 [73]	140 (95)	2.4‐mg/wk semaglutide	E + D	68	−15.3	−5.3 (34.6)	Multi‐site randomized, double‐blind, placebo‐controlled study design.

Abbreviations: BIA, bioelectrical impedance analysis; d, day; D, diet; DXA, dual‐energy x‐ray absorptiometry; E, exercise; FFM, fat‐free mass; kg, kilogram; mg, milligram; SMM, skeletal muscle mass; TBW, total body water; wk, week.

^a^ Includes any instruction or guidance regarding exercise and diet intended to change the behavior of participants.

^b^ Factor of 1.04 used to convert volume (liters) to mass (kilograms).

^c^ Study only reported results from pooled data.

^d^ Lean mass loss is only reported as a percentage (lean mass change in kg not available); total weight lost, lean mass percentage change, and baseline lean mass were used to determine lean mass lost in kg and percent of weight lost from lean mass.

^e^ Lean mass change only reported as percentage change and change in ratio of fat mass to lean mass pooled across dosages (lean mass changes not reported for individual doses); change in lean mass in the table is therefore approximated and was calculated from average weight lost across doses and reported change in fat mass to lean mass ratio pooled across dosages.

**TABLE 3:** Studies reporting impact of FDA‐approved anti‐obesity medications on loss of compartment termed “fat‐free mass.”

Body composition measurement	Study	Total *N* (*N* intervention group(s))	Anti‐obesity medication, dosage(s), and frequency	Exercise (E) or diet (D) intervention^a^	Intervention length (weeks)	Weight change (kg)	kg Change in fat‐free mass (% total weight lost)	Study design and comments
Hologics QDR 4500A (Bedford, MA)	Martin et al., 2011 [74]	57 (29)	10‐mg 2×/d lorcaserin	E + D	8	−3.8	−2.4 (63.2)	Randomized, double‐blind, placebo‐controlled, parallel group study design.
Multifrequency BIA model Bodystat 1500 (Bodystart Ltd., Douglas, UK)	van Eyk et al., 2019 [75]	50 (24)	1.8‐mg/d liraglutide	—	26	−4.3	−2.4 (55.8)	Randomized, double‐blind, placebo‐controlled study design. Uses frequencies at 5 and 50 kHz. Estimates TBW, intracellular, and extracellular water. Authors used the term lean mass, but it included bone according to the manual.
Lunar iDXA (GE Healthcare)	Grannell et al., 2021 [76]	78 (59)	3.0‐mg/d liraglutide	E + D	16	−12.2	−2.3 (18.9)	Nonrandomized prospective study design.
Inbody720 multifrequency analyzer (Biospace, Seoul, South Korea)	Kwon et al., 2021 [77]	112 (57)	37.5‐mg phentermine HCl	—	12	−6.2	−4.9 (79.0)^b^	Randomized, double‐blind, placebo‐controlled study design. This BIA measures at frequencies of 1, 5, 50, 250, 500, and 1000 kHz and allows for estimation of total, intracellular, and extracellular water.
			Orlistat 120 g 3×/d + phentermine HCl 37.5 mg/d			−6.0	−3.8 (63.3)^b^	

Abbreviations: BIA, bioelectrical impedance analysis; d, day; D, diet; DXA, dual‐energy x‐ray absorptiometry; E, exercise; kg, kilogram; mg, milligram; TBW, total body water; wk, week.

^a^ Includes any instruction or guidance regarding exercise and diet intended to change the behavior of participants.

^b^ To maintain consistency of results reported, change in fat‐free mass was obtained from reported [median baseline median weight—12 week median weight] because reported fat‐free mass change was reported as mean change and was greater than total weight change.

**TABLE 4:** Studies reporting impact of FDA‐approved anti‐obesity medications on loss of compartment termed “muscle mass” or “skeletal muscle mass.”

Body composition measurement	Study	Total *N* (*N* intervention group(s))	Anti‐obesity medication, dosage(s), and frequency	Exercise (E) or diet (D) intervention^a^	Intervention length (weeks)	Weight change (kg)	kg Change in muscle mass (% total weight lost)	Study comments and limitations
Lunar Prodigy (General Electric Medical Systems, Madison, WI)	Ishoy et al., 2016 [78]	40 (20)	2‐mg/wk exenatide	—	12	−2.2	0.0 (0.0)	Randomized, double‐blind, placebo‐controlled, parallel group study design. Reported on “muscle mass,” which is not a listed variable in GE prodigy output. Unknown whether “muscle mass” was derived or is an incorrectly labelled output variable.
TANITA BC‐420 MA (TANITA BIA Technology, Amsterdam, The Netherlands)	Keskin and Yaprak, 2022 [79]	276 (138)	3.0‐mg/d liraglutide	D	26	−11.3	−1.0 (8.6)^b^	Randomized, placebo‐controlled, parallel group study design. Reported on muscle mass, which included smooth and SMM [80]. This BIA, measures at a single frequency of 50 kHz, does not perform segmented analysis and only measures TBW.
Magnetic resonance imaging of thigh (skeletal muscle volume)	Pandey et al., 2024 [81]	128 (73)	3.0‐mg/d liraglutide	E + D	40	—	−0.3 L or −0.3 kg (−3.2% change in thigh muscle volume)^b^	Secondary analysis of randomized, double‐blind, placebo‐controlled study design. Reported muscle volume only.

Abbreviations: BIA, bioelectrical impedance analysis; d, day; D, diet; E, exercise; kg, kilogram; mg, milligram; wk, week.

^a^ Includes any instruction or guidance regarding exercise and diet intended to change the behavior of participants.

^b^ Factor of 1.04 used to convert volume (liters) to mass (kilograms).

While multiple studies have reported on the impact of AOMs on weight loss and related health outcomes, few have investigated their impact on SMM. No studies have investigated potentially deleterious long‐term effects (e.g., maintenance of reduced weight) of SMM loss secondary to weight loss involving AOMs. A PubMed search conducted on February 24, 2025, using a predetermined search strategy (Table S1) identified relevant studies. Of 412 records retrieved, 22 RCTs were included.

Studies were often not comparable with respect to AOMs used, dosage given, intervention length, body composition compartment reported, or inclusion of a lifestyle modification. This variability makes it challenging to derive a consensus on the amount and significance of SMM lost. Most studies provided varying doses of glucagon‐like peptide‐1 receptor agonist (GLP‐1 RAs), measured FFM, LST, or ULM via DXA, and with intervention periods ranging from 8 to 52 weeks. Fewer studies (*n* = 6) evaluated the impact of non‐incretin AOMs on changes in FFM, LST, or SMM.

## 5 Is the Quantity of SMM Lost by AOMs Different Than With Lifestyle Intervention?

Since all forms of weight loss will result in loss of both fat and FFM, understanding whether AOM‐induced SMM loss is greater or less relative to SMM loss secondary to lifestyle intervention is an important consideration. The most assessed AOM‐dose combinations, 1.8 (*n* = 6) and 3.0 mg (*n* = 6) liraglutide, led to highly variable ULM or FFM losses between 0%–65% [67, 68, 69, 70, 72, 75] and 0%–34% [65, 66, 76, 79], respectively. Meanwhile, lifestyle interventions lead to a much tighter range of losses, between 16% and 33% [49, 50, 82].

**FIGURE 3:**
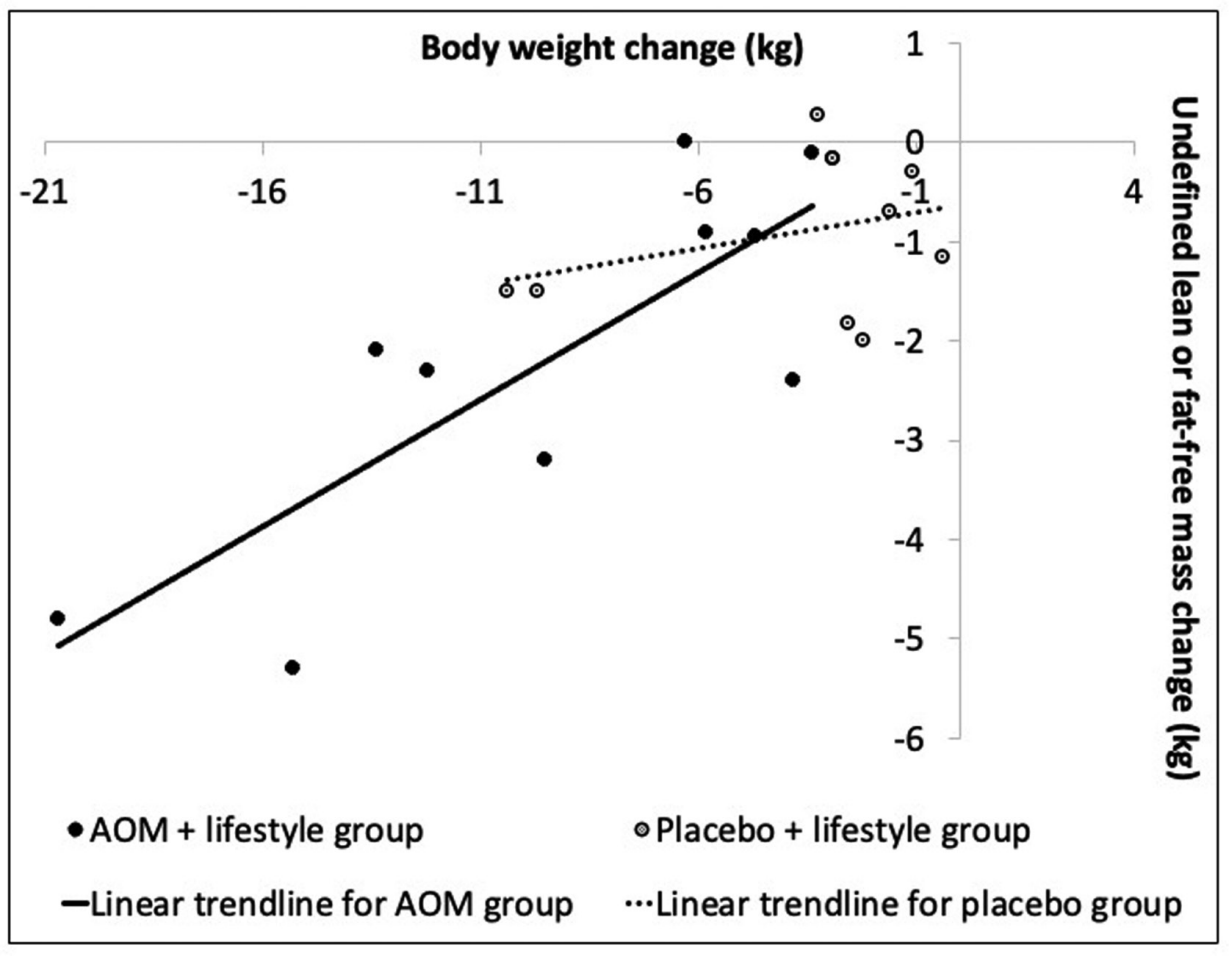
Change in fat‐free mass or undefined lean mass measured via DXA or BIA relative to body weight change in AOM intervention group and placebo group, both undergoing lifestyle intervention. Only studies (*n* = 9) that specified a diet or exercise prescription for both AOM and placebo groups are included. Fidler et al. [64] had two dosage groups of lorcaserin, and thus represent separate points on the graph (designated with *). Abbreviations: AOM, anti‐obesity medications; BIA, bioelectrical impedance; DXA, dual‐energy x‐ray absorptiometry; kg, kilograms.

Given the heterogeneity in study designs, comparing lifestyle intervention and AOM required examination of each study to determine the SMM‐related body composition compartment reported (most reported on ULM or FFM), and whether studies included an additional lifestyle intervention to placebo and AOM groups. In studies using DXA [17, 64, 65, 66, 67, 73, 74, 76] and BIA [60] to estimate FFM or ULM where both placebo and intervention groups received guidance or instruction to change lifestyle behaviors (i.e., physical activity or diet), placebo groups all lost FFM or ULM, albeit one that reported a gain [17]. Similarly, intervention groups always lost FFM or ULM, with the exception of one study (Figure 3) [66]. Several studies were highly nonspecific regarding diet and physical activity interventions and were thus not included.

An alternate approach is to compare FFM or ULM between placebo and AOM groups exposed to the same lifestyle intervention. Three studies reported within‐group changes in FFM or ULM but did not report on differences in FFM or ULM change between groups [17, 65, 73]. One study providing 10 mg of lorcaserin twice daily resulted in significant losses of ULM, with no significant loss at 10 mg once daily [64]. Compared to a placebo group, one study reported significantly greater losses of FFM with treatment of lorcaserin [74]. Four studies assessed various doses of liraglutide [66, 67, 72, 76], and one evaluated phentermine HCl [60] and found no differences in FFM or ULM change between placebo and AOM groups, both undergoing lifestyle intervention. One of these studies showed a significant difference in FFM loss before, but not after, adjusting for total weight lost [76].

One study evaluated changes in SMM using neck‐to‐knee MRI following supplementation with 3.0‐mg/d liraglutide for 40 weeks, and reported that ~3% of weight lost was from SMM [59]. A secondary analysis of the same study reported a 3% loss of thigh muscle volume assessed by MRI [81]. Interestingly, this study included a diet and exercise component as part of the intervention and reported similar SMM losses (~2% loss of thigh muscle mass assessed via MRI) to interventions including diet and exercise without pharmacological intervention [51, 83]. While this may suggest that the inclusion of lifestyle components to AOMs may aid in the preservation of SMM, no studies herein included a three‐way comparison that would inform the role of concurrent lifestyle interventions and AOM (i.e., AOM + lifestyle vs. AOM alone vs. placebo + lifestyle) for SMM or SMM‐related body compartments. Additionally, only one study reported on adherence to lifestyle interventions [76].

In summary, available studies suggest that FFM and ULM, NOT necessarily SMM or LST, are lost in proportion to weight loss with the use of AOMs. Only two studies from the same cohort estimated SMM changes with 3.0‐mg/d liraglutide using MRI and found the amount of SMM lost was not different to that observed in lifestyle change‐related weight loss [59, 81]. However, this finding cannot be extrapolated to other AOMs, doses of liraglutide, or intervention lengths. With two exceptions [59, 81], studies do not address the contribution of SMM to observed changes in LST or FFM—rather, LST and FFM reflect the combined changes of SMM, smooth muscle, organs, extracellular fluid, and, in some cases, bone. This reflects a notable gap in the literature.

Given the lack of precision and uniformity noted in the available studies, the following sections may provide some guidance for the design of future studies focused on characterizing SMM including how reporting could be improved to increase clarity and precision.

## 6 Body Compartment Terminology and Reporting

Comparison of study results was difficult due to the reporting of LM but not defining whether LST (no bone) or FFM (includes bone). Additionally, the terms FFM and LM are often used interchangeably when referring to the nonfat compartment with the assumption that they are equivalent. Most studies (*n* = 16/22) returned in the search obtained FFM or “LM” using DXA, and few studies reported on both FFM and LM. Remarkably, few studies made the distinction between whether LM was LST or FFM. Further, it was difficult or impossible to determine whether bone mass was included if the DXA model used was not reported [62, 64, 72, 73].

This imprecise reporting impacts our ability to draw conclusions regarding AOM‐induced changes in SMM, LST, or FFM. For example, two studies provided 37.5‐mg phentermine for 12 weeks [60, 77], yet reported very different changes in body composition. While this could be explained partially by differences in study design or BIA InBody models (230 vs. 720), it is unknown if reported SMM‐related components were consistent across studies. One study used the term LM and did not describe its components [60], while the other was defined as FFM [77]. Other studies reported different “SMM proxies” (i.e., LM vs. FFM) and did not define the term LM [17, 59, 60, 63, 64, 65, 66, 67, 68, 69, 70, 71, 72, 73] or use terminology consistent with the operator manual [61].

## 7 Body Hydration and Standard Procedures

Assumptions related to hydration levels (i.e., 73% of FFM in adults is water) embedded in two‐ and three‐compartment models where TBW is not being measured can contribute to error within and outside the context of obesity [29]. BIA estimates FFM and then derives fat mass (two‐compartments: using the formula: Fat = Body Mass − (Total Body Water / 0.73)), while simply speaking, DXA estimates fat mass, LST, and bone mineral content (BMC) (three‐compartments). Since both methods assume hydration levels of fat mass and FFM, they rely heavily on the accuracy of the hydration assumption and are susceptible to errors when variations in hydration apply. In the context of overweight and obesity, DXA and BIA have been shown to overestimate FFM compared to three‐ and four‐compartment models that include TBW estimation [29]. This overestimation may be partly due to the increased ratio of extracellular water to intracellular water and increased body hydration observed in individuals with obesity and following weight loss [35, 84].

Standardized measurement conditions (i.e., time of day, restraint from physical activity or alcohol, empty bladder, and fasting) minimize confounding of results related to hydration, for DXA and BIA, especially when the study design involves longitudinal or repeated measures, when measuring body composition changes during weight loss. Variations in DXA and BIA measurements under standardized (overnight fasted, rested, and hydrated) versus nonstandardized (afternoon and nonfasted) conditions were explored in male athletes, while a 500 g meal (consisting of 62‐g carbohydrate, 36‐g protein, 10‐g fat, and 340‐mL fluid) had little effect on DXA‐measured FFM and fat mass, a larger meal (500 g plus 1 L of water) caused a moderate increase (1.2 kg) in DXA‐measured FFM. BIA results were influenced by both meals: Specifically, the time of day and non‐fasting state (participants were measured in the afternoon after ad libitum food and fluid intake) increased FFM by 2.7 kg [85].

Most AOM studies (20/22) used measures to estimate SMM susceptible to variations in hydration and did not report any standardized procedures. The most commonly reported standardization procedure was that participants were fasted [65, 66, 68, 70, 71, 73, 79], with only one study reporting the length of the fast (12 h) [66]. No study reported that participants voided immediately prior to their measurement. Only one study described what participants wore during the test [74], and another reported that participants would follow a standard diet and refrain from strenuous exercise or alcohol 24 h prior to the procedures [65]. Few studies reported the time of day for measurements, with morning having the least variability.

Even with standardization procedures in place for BIA and DXA, assessment of hydration may be warranted in studies involving AOMs, as dehydration may occur as an unintended consequence of AOM use. No studies from the search herein determined hydration status in their study participants before measurements. The most common adverse event reported with the use of GLP‐1 RAs is mild to moderate gastrointestinal symptoms such as nausea, vomiting, diarrhea, and constipation [86]. Depending on the severity and duration, these symptoms reflect perturbations in hydration regulation due to fluid/electrolyte loss or lack of intake. Studies to date have not considered the potential error in body composition measures that may manifest from these adverse events.

Assessment of hydration status could ameliorate shortcomings of methods reliant on adequate hydration. Measures of plasma/serum or urinary osmolality, urine color, and urine specific gravity could serve as relatively accurate, cost‐effective, and low‐burden (especially since blood samples are often collected in AOM clinical trials for other purposes) assessments of hydration. Neutron activation and stable isotope dilution methods are highly accurate but lack feasibility in clinical settings due to limited availability, expense, and prolonged analysis time [87].

## 8 Assumptions Regarding Proportions Between SMM and FFM or LST

Estimation of SMM using indirect measures (i.e., TBK, DXA, and BIA) requires knowledge of the proportion of FFM that is SMM and requires careful analysis in the context of obesity treatment. The amount of FFM that is SMM may not remain constant at reduced weights. For individuals with obesity undergoing bariatric surgery, SMM was 47% and 50% of FFM in women and men, respectively. One‐year post‐bariatric surgery, SMM for women and men was 41% and 45% of FFM, and remained reduced (compared to presurgery) 5‐year postsurgery [27]. Individuals undergoing bariatric surgery from this study had significantly greater FFM compared to controls at Years 1 and 2 postsurgery. However, when FFM was reviewed based on its sub‐components, SMM (by MRI) was significantly lower at Years 1 and 5, while trunk organs were increased at Years 1, 2, and 5 postsurgery compared to controls who did not lose weight [88]. These results indicate that, although SMM is the largest component of FFM, change in SMM during weight loss is not necessarily proportional to FFM change.

Bone mineral is an important component of FFM that may change during weight loss [89]. Despite the numerous health benefits of weight loss in individuals with obesity, weight loss via caloric restriction without weight‐bearing exercise results in increased biomarkers of bone breakdown and reduced hip BMD, increasing the risk of fracture in these individuals [90]. Few studies thus far have investigated the impact of GLP‐1 RAs, other combined incretin therapies, and AOMs on BMD or BMC in persons with overweight or obesity [89, 91]. GLP‐1 RA intervention over 52 weeks (i.e., exenatide and dulaglutide) was protective against BMD loss (a gain was reported for exenatide) in individuals with T2DM [92]. Similarly, glucose‐tolerant women with obesity receiving liraglutide for 52 weeks to promote weight loss maintenance had four times lesser BMC losses in the arms and legs and increases in a bone formation marker compared to a placebo group (neither group exhibited BMD changes during weight maintenance) [93]. Given the likelihood that GLP‐1 RAs have unique effects on bone turnover, density, or content [91], bone‐specific investigations independent of SMM studies are required with appropriate nomenclature to inform the reader (i.e., FFM includes both SMM and BMC; LST excludes BMC and is largely a surrogate for SMM by DXA).

## 9 DXA‐Specific Limitations

Explanations for the wide variability in losses of LST or FFM may extend beyond basic study characteristics such as reported body compartment, intervention length, variations in hydration, and lifestyle modifications. X‐rays used by DXA may be attenuated by greater trunk depth resulting from excess trunk fat mass—DXA may overestimate bone by up to 20.5% and fat by up to 11.1% when increasing thicknesses of lard are added to a section of beef femur [94]. While newer DXA machines typically have scan modes that account for body (trunk) thickness, these modes are either automatically determined by the software or can be selected by the technician. Similarly, newer DXA machines have automated segmental analyses that allow for the identification of regions of interest (ROIs) like visceral fat or individual leg or arm fat and FFM. These selections (whether automated or self‐selected) may represent significant sources of error for body composition assessment due to inconsistent ROI selection between manufacturers and devices and, for individuals with obesity, varying body fat distribution (e.g., adipose tissue extending laterally vs. increasing thickness) [95].

Limited field of view is a less appreciated limitation of DXA that applies to all studies herein. Individuals wider than 60–67 cm (machine dependent) [96, 97] are either excluded from the study, or when included, DXA provides an inaccurate estimation of soft tissues due to part of the body being outside the scan field of view. These individuals are at greater risk for many of the cardiovascular and metabolic comorbidities of obesity and are thus a population that would benefit from greater weight losses that can be achieved with AOMs, compared to lifestyle intervention [98].

The DXA software provides an option for estimating whole‐body composition using a half‐body scan [99] for persons whose body size exceeds the limits of the scanner field of view. However, the accuracy of this approach cannot be validated in persons whose body size exceeds the field of view. Moreover, study results from persons who exceed the field of view at baseline but fit within the field of view following treatment are confounded for calculating changes in all body composition variables. Out of 18 studies reporting the use of DXA to explore changes in FFM or LST with the use of AOMs, one reported the use of a half‐body scan [76], one reported exclusion [17], and the others did not report on methods for individuals whose size extended beyond the field of view.

## 10 Does Proportion of Lean or FFM Increase After Weight Loss From AOMs?

**FIGURE 4:**
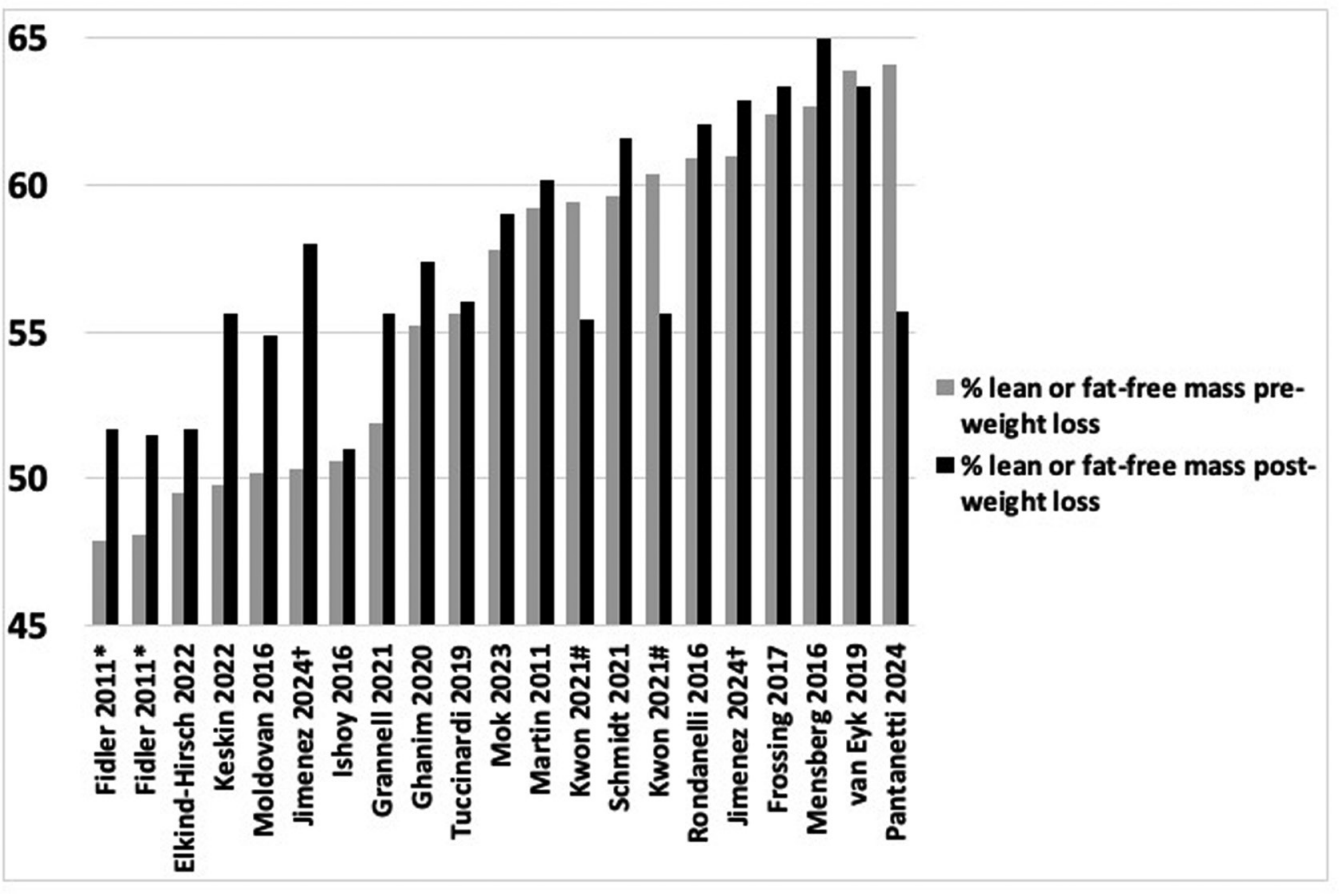
Percent undefined lean or fat‐free mass relative to total body weight before and after weight loss by study group. Studies are shown when percent undefined lean or fat‐free mass could be derived from aggregate data reported in the manuscripts (*n* = 16). Studies are shown in rank order by baseline percent undefined lean or fat‐free mass. Fidler et al. [64]* and Kwon et al. [77]# had multiple intervention groups (e.g., multiple doses of AOM), so group changes in mass are shown separately. No study evaluated statistical significance of differences between pre and post proportions.

The change in percent of body weight that is ULM or FFM is sparsely reported [62, 73, 75]. Percent ULM or FFM was, on average, 55.9% at baseline and 57.4% at post‐weight loss (Figure 4). No studies tested the significance of changes in this proportion from pre‐ to post‐weight loss. However, one study providing various doses of naltrexone/bupropion for 52 weeks reported a significant treatment effect on change in lean‐to‐fat mass ratio (time effect not assessed) [62]. To understand if body composition improves with AOM‐induced weight loss, future studies should report proportional change of LST or FFM to body weight or fat mass lost and evaluate the statistical significance of this change between groups and over time.

## 11 Towards the Future: AOMs Under Development to Preserve SMM

With increased understanding of the importance of SMM to health and the development of drug therapies for muscular atrophy from degenerative conditions and aging, there is interest in developing AOMs to optimize fat mass loss while preserving SMM during weight loss [100, 101]. Specifically, combinations of semaglutide (i.e., Wegovy, Ozempic, and Rybelsus) and tirzepatide (i.e., Zepbound and Mounjaro) with drugs that target various biological pathways involved in the anabolism or catabolism of SMM are currently being explored.

Several of these drug combinations are currently undergoing phase 2 testing, with promising preclinical and preliminary results: The monoclonal antibody that blocks antagonists of muscle hypertrophy (myostatin and activin A), bimagrumab, combined with semaglutide, elicited a 10% increase in LST while simultaneously decreasing fat mass in obese mice [102]. In individuals with obesity and type 2 diabetes, bimagrumab without semaglutide led to 6.5% weight loss, with 29% from LST by DXA [16]. Another drug therapy that inhibits myostatin and activin A, trevogrumab and garetosmab, is being combined with semaglutide in a phase 2 clinical trial. In male cynomolgus monkeys, results showed increased loss of fat mass (−43.9% vs. −25.1%) and a gain in ULM (+6.2% vs. −2.5%) with the combined therapy compared to semaglutide alone over 20 weeks [103]. Enobosarm is being combined with semaglutide as it promotes muscle hypertrophy through modulation of androgen receptors and has demonstrated increases in ULM and physical function in older adults [104]. An ongoing phase 2 trial combines azelagrag, an apelin RA, with tirzepatide. In mice, this combination has shown greater overall weight loss and preservation of LST, such that percent LST (69%) was greater than in those taking tirzepatide alone (60%) and similar to lean controls (70%) [105]. As these therapies develop and companies strive to obtain FDA approval with claims involving retention of SMM during weight loss, it is imperative that limitations be considered when selecting a measurement method, and an appropriate study design is selected.

## 12 Conclusions

AOMs serve as a promising mode of weight loss and post‐weight loss maintenance, given the greater amounts of weight loss possible compared to lifestyle intervention and with numerous studies showing cardiometabolic improvements from their use [17, 63, 67, 71, 77]. There is a specific need to determine how AOMs impact the maintenance of SMM, as it is highly related to mortality and morbidity [7]. Available data from randomized, placebo‐controlled studies are highly heterogeneous (i.e., differing intervention lengths, doses, and medications) and have used SMM estimation techniques with limitations for application in populations with obesity. Notably, these techniques provide estimates of different body composition compartments: SMM, muscle mass, LST, and FFM do not represent the same tissues. There is a critical need for greater precision and depth of understanding when selecting a measurement method, describing body compartments, and intervention‐induced changes in these compartments.

**FIGURE 5:**
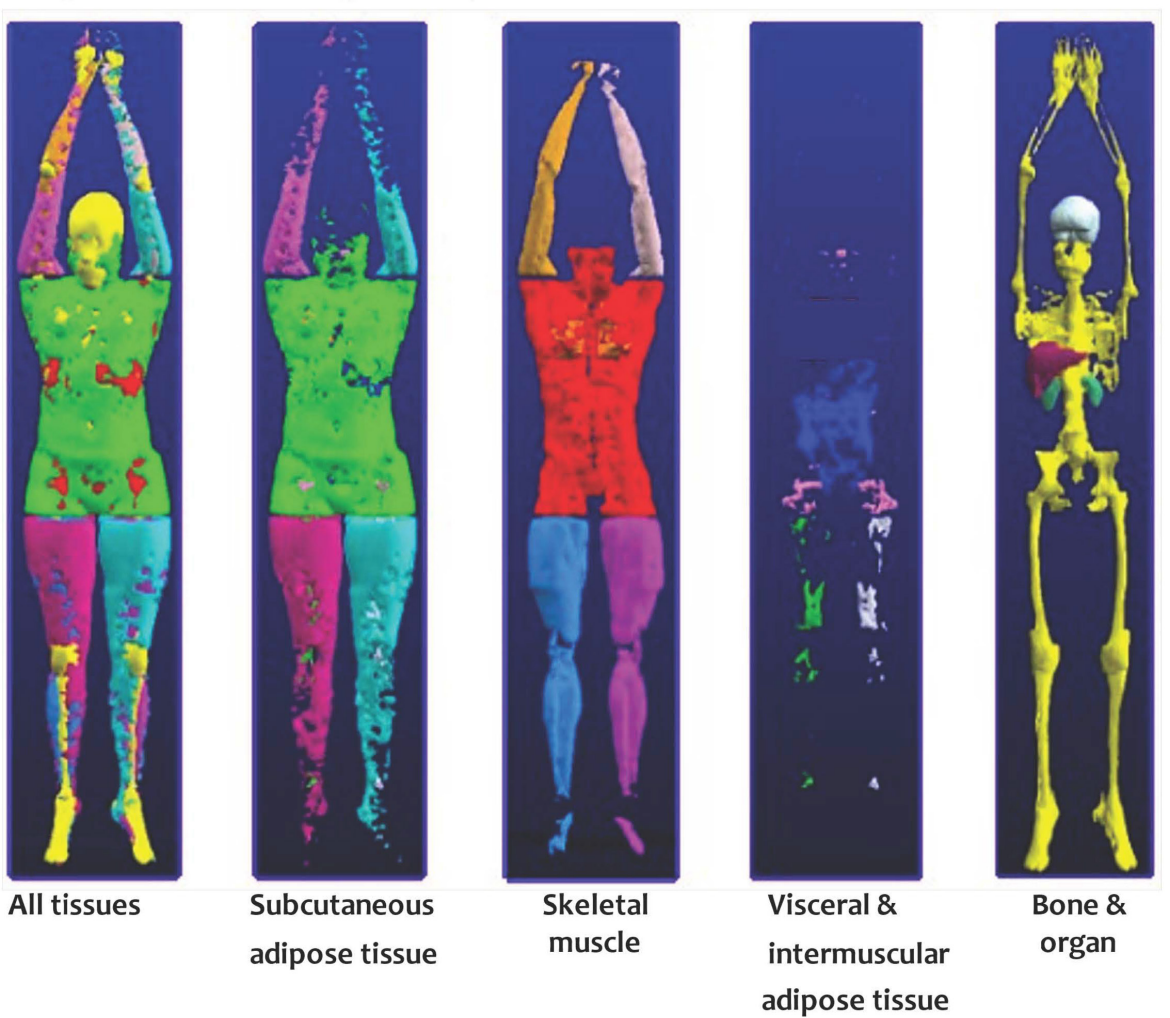
Whole‐body magnetic resonance imaging—shown are three‐dimensional reconstructions of segmented tissue and organ compartments. Image adapted from Shen et al. with permission [106].

Future studies should aim to incorporate more robust estimations of SMM, such as whole‐body MRI [13, 27], which has the added benefit of allowing for quantification of other tissues susceptible to changes by AOMs including subcutaneous, visceral, intermuscular, and liver fat (Figure 5) [106]. Given the cost‐prohibitive nature of these assessments, however, estimations derived via DXA and/or multifrequency BIA could be improved by standardized procedures, measuring hydration status prior to measurement, and use of appendicular SMM variables. Including outcomes of physical function could be informative from a “SMM function” perspective, since statistically significant differences in SMM, FFM, or LST may not be clinically relevant. Given the lack of homogenous interventions with robust measures of physical function or strength [107], currently, no consensus can be reached on the impact of AOMs on SMM function. Relatedly, studies should address the utility of concurrent diet and exercise intervention with AOMs for maintenance of SMM and improvements in physical function by comparing AOM only versus AOM plus lifestyle interventions. Future studies should consider how to implement a more personalized approach, wherein interactions between AOMs and baseline characteristics of patients (e.g., SMM, physical function, insulin sensitivity, and glucose homeostasis) are considered.

## Author Contributions

Dympna Gallagher conceived the concept. Arden McMath performed the literature review and drafted the paper. All authors critically reviewed manuscript drafts and approved the final manuscript. Dympna Gallagher had primary responsibility for final content.

## Funding

This work was supported by the National Institutes of Health (P30‐DK26687, T32‐DK007559 and K26‐DK138418).

## Conflicts of Interest

The authors declare no conflicts of interest.

## Supporting information


**Table S1:** PubMed search strategy.

## Data Availability

Data sharing not applicable to this article as no datasets were generated or analysed during the current study.
