# Supplementary material for: Understanding Impact of Anti‐Obesity Medications on Skeletal Muscle Mass Change Is Confounded by Measurement Methods
Source: Obes Rev. 2025 Nov 24;27(4):e70041. doi: 10.1111/obr.70041 (PMC13008602; doi:10.1111/obr.70041)
Supplement: Supplementary file 1 — Table S1: PubMed search strategy. [file OBR-27-e70041-s001.docx]

**Supplemental Table 1.** PubMed search strategy.

| ((incretin mimetic*[Title]) OR (GLP-1 receptor agonist [Title]) OR (GLP-1 agonist [Title]) OR (Wegovy[Title]) OR (Zepbound[Title]) OR (Ozempic[Title]) OR (Liraglutide[Title]) OR (Semaglutide[Title]) OR (Orforglipron[Title]) OR (Tirzepatide[Title]) OR (CagriSema[Title]) OR (Survodutide[Title]) OR (Mazdutide[Title]) OR (Retatrutide[Title]) OR (Danuglipron[Title]) OR (Cagrilintide[Title]) OR (PYY 1875[Title]) OR (Efinopegdutide[Title]) OR (Pemvidutide[Title]) OR (AMG 133[Title]) OR (Dapiglutide[Title]) OR (S-309309[Title]) OR (CT-996[Title]) OR (amylin agonist[Title]) OR (AZD6234[Title]) OR (ZP8396[Title]) OR (HM15136[Title]) OR (NNC0165-1562[Title]) OR (Y-14[Title]) OR (VK2735[Title]) OR (VK2735[Title]) OR (SCO-094[Title]) OR (CT-388[Title]) OR (Amycretin[Title]) OR (Dacra QW II[Title]) OR (HM15211[Title]) OR (NNC0247-0829[Title]) OR (JNJ-9090/CIN-109[Title]) OR (SCO-267[Title]) OR (ZP6590[Title]) OR (GIP receptor agonist[Title]) OR (GIP agonist[Title]) OR (amylin receptor agonist[Title]) OR (amylin agonist[Title]) OR (glucagon receptor agonist[Title]) OR (glucagon agonist[Title]) OR (PYY receptor agonist[Title]) OR (PYY agonist[Title]) OR (GDF15 analogue[Title]) OR (phentermine[Title]) OR (Lomaira[Title]) OR (Adipex[Title]) OR (Anorectic*[Title]) OR (lorcaserin[Title]) OR (Belviq[Title]) OR (APD-356[Title]) OR (naltrexone[Title]) OR (bupropion[Title]) OR (Contrave[Title])) AND (randomized[Title/Abstract]) AND (placebo[Title/Abstract]) AND ((weight[Title/Abstract]) OR (mass[Title/Abstract])) NOT (review[Publication Type]) NOT (mice[Title]) NOT (mouse[Title]) NOT (murine[Title]) NOT (rodent[Title]) NOT (systematic review[Publication Type]) NOT (Books*[Publication Type]) NOT (children[Title]) NOT (adolescents[Title]) NOT (in vitro[Title]) NOT (phase 1[Title]) |
| --- |
